# Treatment of Giardiasis after Nonresponse to Nitroimidazole

**DOI:** 10.3201/eid2010.140073

**Published:** 2014-10

**Authors:** Eyal Meltzer, Tamar Lachish, Eli Schwartz

**Affiliations:** The Chaim Sheba Medical Center and the Center for Geographic Medicine, Tel Hashomer, Israel (E. Meltzer, E. Schwartz);; Tel Aviv University, Tel Aviv, Israel (E. Meltzer, E. Schwartz);; Shaare Zedek Medical Center, Jerusalem, Israel (T. Lachish)

**Keywords:** Giardia, nitroimidazole, metronidazole, albendazole, quinacrine, nitazoxanide, paromomycin, giardiasis, Israel, parasites, treatment failure

## Abstract

During January 2008–October 2013, a total of 12 cases of giardiasis at the Chaim Sheba and Shaare Zedek Medical Centers, Israel, did not respond to nitroimidazole; 83.3% were associated with travel and 33% with immunoglobulin deficiency. Among 110 published cases, the most effective treatment was quinacrine (efficacy 90%–100%), but its availability is limited.

*Giardia lamblia* are protozoan parasites distributed globally and mostly transmitted by close contact and consumption of contaminated water or food. Giardiasis occurs in industrialized nations (*1*); occasional waterborne outbreaks have been reported in North America and Europe (*2*,*3*). However, the prevalence of giardiasis is greater in developing countries, >50% among children from many locales (*4*). Among travelers, especially those traveling to developing countries, *G. lamblia* infection is also common; according to the GeoSentinel registry, it is the most frequently diagnosed gastrointestinal pathogen (*5*).

The utility of offering antigiardia medications to patients in areas of giardiasis hyperendemicity has been called into question unless symptoms are severe. Conversely, treatment administered to patients in areas of low giardiasis endemicity (such as ill returning travelers) usually leads to cure (*6*). Since the introduction of metronidazole in 1959, nitroimidazoles have been the main treatment for giardiasis. However, a recent meta-analysis of clinical trials has shown that nitroimidazole treatment fails for 10%–20% of giardiasis patients (*7*); optimal treatment in such cases is not defined, and some agents (e.g., quinacrine) are often unavailable.

## The Study

During January 2008–October 2013, a total of 12 cases of nitroimidazole treatment failure (parasitologically confirmed by either microscopy or antigen testing of fecal samples after nitroimidazole therapy) were seen at the Center for Geographic Medicine and Tropical Diseases at Chaim Sheba Medical Center, Tel Hashomer, and the Tropical Disease Clinic at the Shaare Zedek Medical Center, Jerusalem, Israel. All patients were symptomatic.

Median patient age was 25.0 years (interquartile range 23.7–35.0, mean ± SD 30.4 ± 11.7 years), and 75% of patients were male (Table 1). Of the 12 cases, 10 (83.3%) occurred after travel to developing countries; 8 patients had traveled to Asia (India and/or Thailand) and 2 to Latin America. Of 11 patients evaluated for immune deficiency, 4 (36.4%) had low immunoglobulin levels; of these, 1 patient had pan-hypogammaglobulinemia and 3 had IgA deficiency.

**Table 1:** Management of giardiasis nonresponsive to nitroimidazole treatment, Israel, January 2008–October 2013*

Patient age, y/sex	Travel history	No. failed nitroimidazole courses	Immunologic assessment	Alb	Nitaz	Quin	Paromo
24/M	Israel	9	Pan-hypogammaglobulinemia	Failed†	Failed	NA	NA
35/M	Latin America	3	IgA deficiency	Failed	Failed	Cured	NA
61/M	Israel	1	IgA deficiency	Cured	NA	NA	NA
23/M	Latin America	1	IgA deficiency	Cured‡	NA	NA	NA
25/M	Thailand	3	Normal	Failed	Failed	Cured	NA
45/M	India	3	Normal	Failed	Cured	NA	NA
31/F	India	2	Normal	Failed	NA	NA	Cured‡
26/M	India	1	Normal	Failed	NA	NA	Cured
23/M	India	1	Normal	Cured	NA	NA	NA
24/M	Thailand	1	Normal	Cured	NA	NA	NA
25/F	India	2	Normal	Cured‡	NA	NA	NA
23/F	India, Thailand	1	ND	Cured†	NA	NA	NA

*Alb, albendazole; NA, not administered; ND, not done; nitaz, nitazoxanide; paromo, paromomycin; quin, quinacrine. †Combination albendazole–tinidazole treatment. ‡Patient was clinically asymptomatic but did not provide a posttreatment fecal sample.

The median number of failed courses of nitroimidazole was 2.5 (interquartile range 1–3, mean ± SD 2.7 ± 2.5 courses). All 12 patients received albendazole as a second line of treatment. Of 10 patients for whom complete parasitologic data were available, 4 (40%) experienced cure. Of 6 patients for whom albendazole failed, 4 received nitazoxanide. Of these, nitazoxanide led to cure for 1 (25%); subsequent treatment with quinacrine led to cure for 2, and treatment with paromomycin led to cure for the other.

## Conclusions

Despite the high prevalence and global reach of giardiasis, reports of the treatment approach for cases that fail to respond to nitroimidazole are scarce. A review of the literature identified only 12 reports describing treatment outcomes for giardiasis patients after nitroimidazole treatment failure: 7 case series and 5 isolated case reports (Technical Appendix). Together with the series reported here, 110 cases have been described (Table 2). Before 1994, only 2 (2%) cases had been reported; during 1994–2003 a total of 29 (26%) cases, and during 2004–2013 a total of 79 (72%) cases.

**Table 2:** Reports on management of giardiasis nonresponsive to nitroimidazole treatment worldwide, 1962–2013*

Country and publication year	No. cases	No. patients who visited developing countries	No. cured/no. treated (% cured), by treatment type						
			Alb	Paromo	Nitaz	Alb + nitroimid	Quin	Quin + nitroimid	Paromo + nitroimid
Israel, 2014 (this study)	12	10	3/9 (33.3)	1/1 (100)	1/4 (25.0)	1/2 (50.0)	2/2 (100)	–	–
Spain, 2014	3	3	–	–	–	–	3/3 (100.0)	–	–
Spain, 2013	14	14	0/2	0/4	–	–	14/14 (100)	–	–
Spain, 2010	10	8	0/2	0/3	–	1/1 (100)	–	4/4 (100)	2/2 (100)
Norway, 2008	38	0	–	3/6 (50.0)	–	30/38 (78.9)	–	3/3 (100)	–
United States, 2001	5	0	0/2	1/3 (33.3)†	–	–	–	5/5 (100)	–
France, 2000	3	0	1/3 (33.3)	–	–	–	–	–	–
Italy, 1995	20	0	2/10 (20.0)	–	–	9/10 (90.0)	–	–	–
Single case reports, 1962–2008‡	5	1	0/4	–	1/1 (100)	1/2 (50.0)	0/2	2/2 (100)	–
Total	110	36	6/32 (18.7)	5/17 (29.4)	2/5 (40.0)	42/53 (79.2)§	19/21 (90.5)	14/14 (100)	2/2 (100)

*Complete reference information available in the online Technical Appendix (http://wwwnc.cdc.gov/EID/article/20/10/14-0073-Techapp1.pdf). Alb, albendazole; nitaz, nitazoxanide; nitroimid, nitroimidazole; paromo, paromomycin; quin, quinacrine; –, drug not used. †Used in combination with bacitracin. ‡Single case reports from France, Switzerland, Canada, Saudi Arabia, and Thailand. §p<0.001 by Fisher exact test for comparison with alb monotherapy.

For the group of 110 patients, regimens combining (the already failed) nitroimidazoles with other agents led to a cure rate of 86.1% (Table 2). The most prominent effect of nitroimidazole combination was found for albendazole; monotherapy resulted in a cure rate of only 18.7%, whereas combination with nitroimidazole led to a cure rate of 80.8% (Fisher exact test p<0.001). Consistently, the best results were achieved with quinacrine; 90.5% and 100% of patients were cured with monotherapy and in combination with nitroimidazole, respectively. Only 2 (40%) of 5 and 5 (29.4%) of 17 patients who experienced nitroimidazole treatment failure were cured after treatment with nitazoxanide and paromomycin monotherapy, respectively.

Thus, most antiprotozoal agents seem to perform poorly after nitroimidazole has failed. Clearance of giardia after nitroimidazole treatment failure was achieved for <20% of patients who received albendazole and <30% of patients who received paromomycin, despite reported cure rates of 80% (*7*) and 90% (*8*), respectively, for these drugs when used as primary regimens. Clinical trials have reported that the giardia clearance rate after primary monotherapy with nitazoxanide also approached 80% (*9*). Its role in cure after nitroimidazole treatment failure has not been established, but in the few cases found in our search, the giardiasis cure rate after nitazoxanide treatment was only 40% (Table 2). Thus, nitroimidazole treatment failure might actually be an indicator for multidrug-resistant *G. lamblia *strains.

Whether nitroimidazole treatment failure reflects pathogen resistance or defective host defenses is not clear. In our case series and others, immunoglobulin deficiency was common in cases of nitroimidazole treatment failure. It has been shown in select cases that nitroimidazole therapy in immunoglobulin-deficient patients fails to clear nitroimidazole-sensitive giardia, leading to in vivo emergence of resistant strains (*10*). The role of a host factor might also be evidenced by cases in which giardia are refractory to nitroimidazole in 1 patient but easily eliminated from the patient’s family members, as occurred for a patient in our series. This 35-year old patient was IgA deficient, and his wife and toddler son were asymptomatic carriers of giardia, according to fecal testing; giardiasis was parasitologically cured by metronidazole for the family members but not for the patient, for whom repeated courses of the same treatment and other regimens failed. Similar reports from other case series include a family of 4 who were infected simultaneously with *G. lamblia* while traveling in India; although PCR indicated that the strains from all 4 patients were genetically identical, responses to treatment with tinidazole varied (*11*).

Given the poor performance of other monotherapies, the 90.5%–100% rate of response to quinacrine is remarkable. Shortly after the introduction of metronidazole, randomized trials showed it to be as efficacious as quinacrine (*12*), and metronidazole replaced quinacrine entirely as a giardiasis treatment. After 3 decades, the situation seems to be changing; nitroimidazole treatment failure is increasing, and quinacrine seems to be the best treatment for giardiasis.

It is unfortunate that quinacrine is no longer available through pharmaceutical companies; in some countries, it can be obtained only through compounding pharmacies or not at all. In Israel, for example, quinacrine is practically unavailable; for 1 patient in our series, quinacrine was obtained with the kind assistance of colleagues practicing in a developing country. This case illustrates how pharmaceutical industry neglect of tropical parasitic infections carries a health price tag, even in industrialized countries.

Several sources of bias may pertain to retrospective case series, the only extant source of data on nitroimidazole treatment failure for giardiasis. Case series are sometimes the result of point-source (often waterborne) outbreaks and therefore might be biased by the presence of 1 or a few pathogen strains (*3*). Even in reports in which cohorts are more geographically diverse, numbers of patients are generally small and statistical comparison of treatment outcomes is not possible. Moreover, the choice of antiprotozoal agents is influenced by factors other than effectiveness, such as drug availability and cost, which differ from country to country. However, combining all reported cases as we have done eliminates geographic bias, increases numbers, and makes it possible to offer a more reliable view of the treatment of giardiasis not responsive to nitroimidazole.

Among giardiasis patients, nitroimidazole treatment failure is often associated with failure of antiprotozoal drugs in additional classes and with patient immunoglobulin deficiency. Limited data exist to guide treatment when nitroimidazole fails. However, this review of reported cases suggests for this scenario, quinacrine is highly effective and nitroimidazole–albendazole combination therapy is far superior to albendazole monotherapy. Unfortunately, quinacrine is unavailable in many countries, leaving patients with limited and less reliable therapeutic options.

Technical Appendix References used to create Table 2.
