# Supplementary material for: Treatment of Giardiasis after Nonresponse to Nitroimidazole
Source: Emerg Infect Dis. 2014 Oct;20(10):1742–4. doi: 10.3201/eid2010.140073 (PMC4193167; doi:10.3201/eid2010.140073)
Supplement: Technical Appendix — References used to create Table 2. [file 14-0073-Techapp-s1.pdf]

Article DOI: http://dx.doi.org/10.3201/eid2010.140073

Treatment of Giardiasis after Nonresponse to Nitroimidazole, Israel

Technical Appendix

References Used to Create Table 2

1. Requena-Méndez A, Goni P, Lobez S, Oliveira I, Aldasoro E, Valls ME, et al. A family cluster of giardiasis with variable treatment responses: refractory giardiasis in a family after a trip to India. Clin Microbiol Infect. 2014;20:O135–8. PubMed

2. Muñoz Gutiérrez J, Aldasoro E, Requena A, Comin AM, Pinazo MJ, Bardaji A, et al. Refractory giardiasis in Spanish travellers. Travel Med Infect Dis. 2013;11:126–9. PubMed http://dx.doi.org/10.1016/j.tmaid.2012.10.004

3. Lopez-Velez R, Batlle C, Jimenez C, Navarro M, Norman F, Perez-Molina J. Short course combination therapy for giardiasis after nitroimidazole failure. Am J Trop Med Hyg. 2010;83:171–3. PubMed http://dx.doi.org/10.4269/ajtmh.2010.09-0742

4. Mørch K, Hanevik K, Robertson LJ, Strand EA, Langeland N. Treatment-ladder and genetic characterisation of parasites in refractory giardiasis after an outbreak in Norway. J Infect. 2008;56:268–73. PubMed http://dx.doi.org/10.1016/j.jinf.2008.01.013

5. Nash TE, Ohl CA, Thomas E, Subramanian G, Keiser P, Moore TA. Treatment of patients with refractory giardiasis. Clin Infect Dis. 2001;33:22–8. PubMed http://dx.doi.org/10.1086/320886

6. Lemée V, Zaharia I, Nevez G, Rabodonirina M, Brasseur P, Ballet JJ, et al. Metronidazole and albendazole susceptibility of 11 clinical isolates of *Giardia duodenalis* from France. J Antimicrob Chemother. 2000;46:819–21. PubMed http://dx.doi.org/10.1093/jac/46.5.819

7. Cacopardo B, Patamia I, Bonaccorso V, Di Paola O, Bonforte S, Brancati G. Synergic effect of albendazole plus metronidazole association in the treatment of metronidazole-resistant giardiasis [in Italian]. Clin Ter. 1995;146:761–7. PubMed

8. Kampitak T. Selective immunoglobulin M deficiency in a patient with refractory giardiasis. J Investig Allergol Clin Immunol. 2010;20:358–60. PubMed

9. Sawatzki M, Peter S, Hess C. Therapy-resistant diarrhea due to *Giardia lamblia* in a patient with common variable immunodeficiency disease. Digestion. 2007;75:101–2. PubMed http://dx.doi.org/10.1159/000104728

10. Abboud P, Lemee V, Gargala G, Brasseur P, Ballet JJ, Borsa-Lebas F, et al. Successful treatment of metronidazole- and albendazole-resistant giardiasis with nitazoxanide in a patient with acquired immunodeficiency syndrome. Clin Infect Dis. 2001;32:1792–4. PubMed http://dx.doi.org/10.1086/320751
